# Investigating the Link Between Abdominal Pain, Weight Loss, and IgG4-Related Pancreatitis: A Case Report

**DOI:** 10.7759/cureus.41644

**Published:** 2023-07-10

**Authors:** Shafaat Hussain, Menahil Saeed, Shahzeb Saeed, Shanza Faridi

**Affiliations:** 1 Urology, Colchester General Hospital, Colchester, GBR; 2 Medicine, Islamic International Medical College, Rawalpindi, PAK; 3 Internal Medicine, Army Medical College, Islamabad, PAK; 4 Medicine, Army Medical College, National University of Medical Sciences (NUMS), Rawalpindi, PAK

**Keywords:** lymphoplasmacytic infiltration, igg4 -related disease, corticosteroid therapy, igg4-related pancreatitis, chronic abdominal pain

## Abstract

This case report describes a 55-year-old male who presented with a two-year history of abdominal pain and weight loss, along with recent onset nausea, vomiting, and anorexia. Laboratory tests revealed elevated serum IgG4 levels and anemia, and abdominal CT showed diffuse pancreatic enlargement and multiple retroperitoneal lymphadenopathy. Endoscopic ultrasound-guided fine-needle aspiration of the pancreatic mass revealed dense lymphoplasmacytic infiltration with fibrosis and increased numbers of IgG4-positive plasma cells, consistent with the diagnosis of IgG4-related pancreatitis. Treatment with prednisone led to a significant improvement in the patient's symptoms and laboratory values, with subsequent reduction in the size of the pancreatic mass and retroperitoneal lymphadenopathy. The patient remained asymptomatic at the six-month follow-up visit, and serum IgG4 levels remained within the normal range. This case highlights the importance of considering IgG4-related disease (IgG4-RD) in patients presenting with abdominal pain and weight loss and the potential effectiveness of corticosteroid therapy in managing this condition.

## Introduction

IgG4-related disease (IgG4-RD) is a rare autoimmune condition affecting various body organs, including the pancreas, salivary glands, and lymph nodes. The presence of lymphoplasmacytic infiltration and fibrosis identifies this illness, and it can present with vague symptoms like abdominal pain, fatigue, and weight loss. Since the first description of IgG4-RD in 2003, there has been growing recognition of this disease. It is now considered a critical differential diagnosis for patients with unexplained organ enlargement or fibrosis. Diagnosis is made by analyzing clinical and laboratory data and histological features of the biopsy sample. Elevated serum IgG4 levels are a hallmark of the disease. However, not all patients with IgG4-RD have elevated IgG4 levels, moreover, other conditions can also cause elevated IgG4 levels. Therefore, biopsy remains an essential tool for diagnosing IgG4-RD [1].

Glucocorticoids are the first-line treatment for IgG4-RD and can induce remission in most patients. However, long-term follow-up is necessary to monitor for disease recurrence and the potential development of other autoimmune conditions [2]. Here, we present a case of IgG4-RD involving the pancreas and retroperitoneal lymph nodes. It is crucial to take into account this particular condition as a significant factor when diagnosing patients who exhibit unexplained abdominal pain, weight loss, and elevated IgG4 levels.

## Case presentation

A 55-year-old male presented to the Internal Medicine clinic with a two-year history of abdominal pain and weight loss. The patient reported a recent onset of nausea, vomiting, and anorexia. He denied any history of tobacco or alcohol use and had no significant past medical history. On physical examination, the patient appeared thin and pale. The patient's abdomen was soft but diffusely tender to palpation, with no palpable mass. The rest of his physical examination was unremarkable. The patient's laboratory values showed anemia and elevated serum IgG4 levels (Table 1).

**Table 1:** Laboratory test indicating elevated serum IgG4 and anemia of chronic disease. CBC, complete blood count; WBC, white blood cell; RBC, red blood cell; HCT, hematocrit; MCV, mean corpuscular volume; MCH, mean corpuscular hemoglobin; MCHC, mean corpuscular hemoglobin concentration; TIBC, total iron binding capacity; AST, aspartate aminotransferase; ALT, alanine transaminase; GGT, gamma-glutamyl transferase

Laboratory test	Result	Normal range
CBC		
WBC count	5	4-11 x 10^9^/L
Total RBC	3.9	3.8-5.2 x 10^12^/L
Hemoglobin	10.5	13-18 g/dL
HCT	36	35%-46%
MCV	84	77-95 fl
MCH	27.7	26-32 pg
MCHC	33	32-36 g/dL
Platelets	200	150-400 x 10^9^/L
Neutrophils	41	40%-80%
Lymphocytes	20	20%-40%
Reticulocytes	0.4%	0.2%-2%
Iron studies		
Fasting serum iron	25	40-180 mcg/dL
Serum ferritin	61	50-100 ng/mL
TIBC	310	250-450 mcg/dL
Transferrin iron saturation %	27	25%-35%
Liver function test		
AST	14	12-37 U/L
ALT	16	15-65 U/L
GGT	22	0-55 U/L
Albumin	3.6	3.4-5.0 mg/dL
Total bilirubin	0.3	0.2-1.0 mg/dL
Alkaline phosphatase	56	50-136 U/L
Kidney function test		
Blood urea nitrogen	6	5-25 mg/dL
Creatinine	0.4	0.3-1.4 mg/dL
Uric acid	3.1	2.5-7.0 mg/dL
Additional test		
Triglycerides	82	<150 mg/dL
Serum IgG4	237	<135 mg/dL

**Figure 1:**
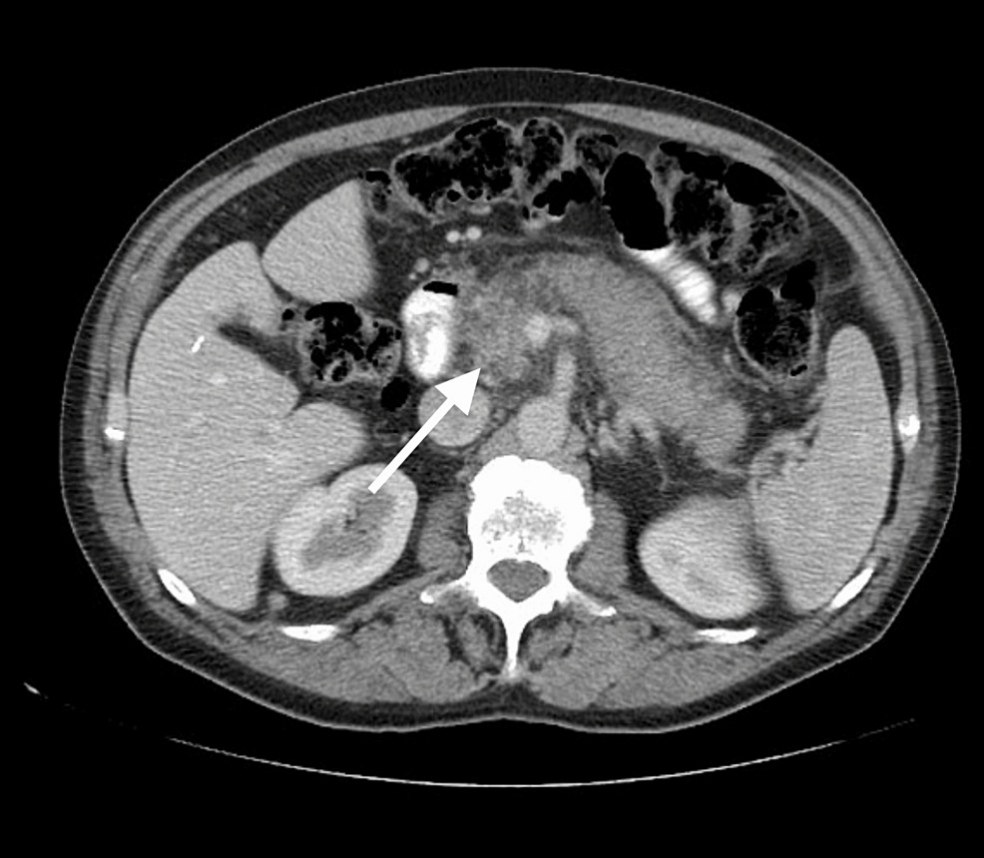
Diffuse pancreatic enlargement and 3-cm mass in the head of pancreas.

Other laboratory values were within normal limits, including liver function tests, triglyceride levels, renal function tests, and electrolytes. Serological tests for hepatitis B and C viruses and human immunodeficiency virus (HIV) were negative. Abdominal CT showed diffuse pancreatic enlargement and multiple retroperitoneal lymphadenopathy (Figure 1). There was no evidence of biliary obstruction or pancreatic duct dilation. The patient was referred to a gastroenterologist for further evaluation.

**Figure 2:**
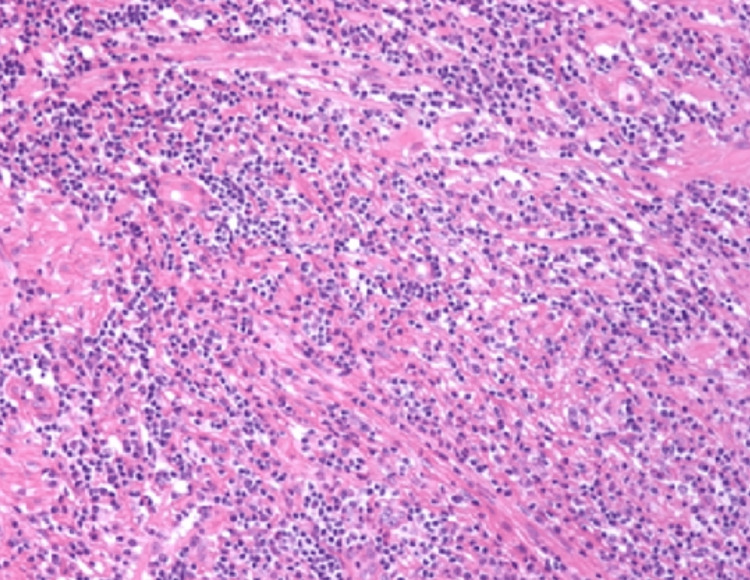
Dense lymphoplasmacytic infiltration with fibrosis and increased numbers of IgG4-positive plasma cells.

Endoscopic ultrasound (EUS) revealed a 3-cm hypoechoic mass in the head of the pancreas. Endoscopic ultrasound-guided fine-needle aspiration (EUS-FNA) of the pancreatic mass was performed, which revealed dense lymphoplasmacytic infiltration with fibrosis and increased numbers of IgG4-positive plasma cells (Figure 2). These findings were consistent with the diagnosis of IgG4-related pancreatitis.

Based on the clinical and histological findings, he was diagnosed with IgG4-RD. The patient was started on prednisone 40 mg/day for four weeks, significantly improving his symptoms and laboratory values. After two weeks of treatment, his serum IgG4 levels decreased to 88 mg/dL, and his hemoglobin levels normalized. We gradually tapered the dose by 5 mg/week over two months and discontinued it entirely. 

At the six-month follow-up visit, the patient remained asymptomatic, and repeat imaging showed a significant reduction in the size of the pancreatic mass and retroperitoneal lymphadenopathies. The serum IgG4 levels remained within the normal range.

## Discussion

IgG4-related disease is a rare autoimmune disease affecting various organs, including the pancreas, salivary glands, lymph nodes, and others. The disease is characterized by lymphoplasmacytic infiltration and fibrosis, which can lead to organ dysfunction and failure. The exact pathogenesis of the disease still needs to be better understood. However, it is thought to involve a dysregulated immune response, with increased production of IgG4 antibodies and activation of inflammatory cells [3].

The diagnosis of IgG4-RD is challenging due to its non-specific clinical presentation. The disease often presents with non-specific symptoms, including abdominal pain, weight loss, and fatigue, which can mimic other medical conditions. Laboratory tests may reveal elevated serum IgG4 levels, but this finding is not specific and can also be seen in other autoimmune diseases, infections, and malignancies. Imaging studies, such as CT, MRI, and positron emission tomography (PET), can show characteristic features of IgG4-RD, such as diffuse organ enlargement, lymphadenopathy, and fibrosis. However, these imaging findings are not specific and can be seen in other medical conditions. The gold standard for diagnosing IgG4-RD is a histological examination of affected tissues, which can show characteristic features of the disease, including dense lymphoplasmacytic infiltration, fibrosis, and increased numbers of IgG4-positive plasma cells. Various methods, including percutaneous biopsy, endoscopic biopsy, and surgical biopsy, can obtain biopsy samples. The choice of the biopsy method depends on the location and accessibility of the affected organ and the medical team's expertise [4].

The treatment of IgG4-RD is primarily based on glucocorticoids, which can induce remission in most patients. The exact mechanism of action of glucocorticoids in IgG4-RD is still poorly understood, but it is thought to involve suppression of the dysregulated immune response and reduction of inflammation and fibrosis. The initial dose of glucocorticoids is typically high, ranging from 30 to 60 mg/day, depending on the severity of the disease and the patient's comorbidities. The dose is then gradually tapered over several months, closely monitoring symptoms and laboratory values. The goal of treatment is to achieve remission of the disease and prevent disease relapse and the progression of fibrosis in affected organs [5].

In addition to glucocorticoids, other immunosuppressive agents, such as azathioprine, mycophenolate mofetil, and rituximab, have been used to treat IgG4-RD, either alone or in combination with glucocorticoids. These agents are typically used in patients who do not respond to glucocorticoids or develop glucocorticoid-related adverse effects. However, their efficacy and safety in treating IgG4-RD still need to be well established, and further studies are required to determine their role in managing the disease [5]. Long-term follow-up is necessary for patients with IgG4-RD to monitor for disease recurrence. Disease relapse can occur in up to 30% of patients after glucocorticoid discontinuation. The risk of relapse is higher in patients with multiorgan involvement, elevated serum IgG4 levels, and complete remission [6].

## Conclusions

Based on the clinical and histological findings, this case report describes a 55-year-old male with a two-year history of abdominal pain and weight loss who was diagnosed with IgG4-related pancreatitis. The patient's symptoms and laboratory values improved significantly with prednisone therapy, and repeat imaging showed a reduction in the size of the pancreatic mass and retroperitoneal lymphadenopathies. The serum IgG4 levels remained within normal at the six-month follow-up visit. This case highlights the importance of considering IgG4-related disease in the differential diagnosis of patients with unexplained abdominal pain and weight loss and the potential effectiveness of glucocorticoid therapy in managing this condition.
